# A short note on measuring subjective life expectancy: survival probabilities versus point estimates

**DOI:** 10.1007/s10198-015-0754-1

**Published:** 2016-01-09

**Authors:** David R. Rappange, Job van Exel, Werner B. F. Brouwer

**Affiliations:** Institute of Health Policy and Management, Erasmus University Rotterdam, Werner Brouwer, Office J8-53, P.O. Box 1738, 3000 DR Rotterdam, The Netherlands

**Keywords:** Life expectancy, Subjective expectations, Survival probabilities

## Abstract

Understanding subjective longevity expectations is important, but measurement is not straightforward. Two common elicitation formats are the direct measurement of a subjective point estimate of life expectancy and the assessment of survival probabilities to a range of target ages. This study presents one of the few direct comparisons of these two methods. Results from a representative sample of the Dutch population indicate that respondents on average gave higher estimates of longevity using survival probabilities (83.6 years) compared to point estimates (80.2 years). Individual differences between elicitation methods were smaller for younger respondents and for respondents with a higher socioeconomic status. The correlation between the subjective longevity estimations was moderate, but their associations with respondents’ characteristics were similar. Our results are in line with existing literature and suggest that findings from both elicitation methods may not be directly comparable, especially in certain subgroups of the population. Implications of inconsistent and focal point answers, rounding and anchoring require further attention. More research on the measurement of subjective expectations is required.

## Introduction

The study of subjective life expectancy (SLE) is important in the context of economic choice behaviour [1], predicting mortality [2] and investment in future health [3]. Such individual subjective expectations may contain information not captured by their objective, actuarial counterparts [4]. Therefore, subjective longevity beliefs are increasingly elicited in order to better understand peoples’ decisions in various life domains, including health.

However, the measurement of SLE is not straightforward. In general, two elicitation approaches can be distinguished: the non-probabilistic and the probabilistic approach.^1^ The first approach concerns the direct measurement of individuals’ subjective estimates of expected lifetime, typically asking for a point estimate. While this method is simple and straightforward to administer, it does not provide information regarding the uncertainty of reaching the specified age [5]. The second elicitation approach asks people for their subjective survival probability (SSP), i.e. their assessment of the probability of surviving to a certain target age. SSPs are used in various large-scale household surveys such as, for example, the Health Retirement Study (HRS) and the Survey of Health, Ageing and Retirement in Europe (SHARE). Research using such data has focused on their accuracy compared to actuarial data, their predictive power for actual mortality, and their relevance in the context of economic decisions [6–8]. SSPs capture uncertainty and allow for computing survival probability distributions, but do not inform directly about SLE, and their elicitation is cognitively demanding [9], leading to inconsistencies [10]. Rounding and focal point answers are common phenomena in both approaches, but remain underexplored [11, 12].

The comparison of results from studies using these different approaches requires the comparison of both elicitation techniques. This helps to understand possible differences between elicitation methods. Moreover, considering the unresolved issues with both approaches, studying different elicitation techniques remains important. Only a few studies have directly related both approaches. Hamermesh [13] first employed both approaches in a single survey, using two unrepresentative samples, and found slightly higher estimates (i.e. 0.5–1 year) when probability estimates were used. Recently, Wu et al. [14] evaluated the consistency of both approaches among Australian respondents aged between 50 and 74 years and indicated that ‘even for those individuals who consistently evaluated their survival probabilities, very few choose life expectancies matching their personal beliefs of survival probabilities’.

In this short note, we report on one of the few studies providing a head-to-head comparison of both elicitation formats administered in one study sample. We show the distribution of responses from both approaches and focus on focal point answers, rounding and the consistency of answers. We compare both formats and relate them to relevant background characteristics of respondents such as health, lifestyle, and age of death of next of kin. Furthermore, we highlight possible consequences of sequential questioning (when eliciting SSPs).

## Methods

### Survey and question formats

A web-based questionnaire was administered to 1223 people, representative for the Dutch population aged between 18 and 65 years in terms of age, gender and education level. The data presented here were collected in the context of a larger study investigating expectations about longevity and quality of life at older age [15], acceptability of less than perfect health states [16], and health state valuations [17].

To get a point estimate of SLE, respondents were asked: “What age do you expect to reach yourself?” Answers could comprise any integer between 0 and 120. This question format has been used before [18, 19]. Then, after introducing the concept of probabilities using two warm-up questions,^2^ respondents were asked: “What are the chances that you will live to be age (*T*) or more?” This question was presented to each respondent for the five target ages (*T*) of 60, 70, 80, 90, and 100 years. Answers could comprise any integer between 0 and 100. The wording is in line with aforementioned household surveys, but we used a range of target ages so that individual subjective survival curves could be estimated [14].

Other relevant components of our survey included questions on demographics (i.e. age, gender, marital status, age of death of next of kin), socioeconomic status (i.e. education, income), health (i.e. having a chronic disease or a severe disorder), and lifestyle (i.e. smoking).

To compare the SLE point estimate to the SSPs directly, we derived a best point estimate from the SSPs by computing the age at which the probability distribution of a respondent intersected 50 %.^3^ We assume that a 50/50 chance of reaching a certain age is a reasonable proxy for what a respondent would report as their SLE and, as such, the most logical comparison with a point estimate.

To further investigate the coherence between the answers to SLE and SSPs questions, we computed a ‘certainty score’ for each individual SLE point estimate in order to ascertain the chance that a respondent would reach his SLE point estimate. For this purpose, we used the probabilities at the surrounding target ages and linear interpolation if the SLE point estimate fell between two target ages (or the probability at a specific target age if the SLE point estimate equalled that target age).^4^

We analysed the correlation between the SLE and SSP point estimates. We used ordinary least square (OLS) regression to investigate variables associated with both point estimates, to explain the computed difference between those estimates, and to assess for which subgroups of respondents the certainty score for the SLE point estimate was closest to 50 %.

## Results

From our initial sample of 1223 respondents we excluded 156 (12.8 %) who completed the online questionnaire in <15 min. This minimal completion time for the questionnaire was determined on the basis of a pilot-test of the questionnaire. Next, we selected the respondents who answered all SSP questions for age 60 and above, i.e. those aged between 20 and 59 years (*n* = 878).^5^ For reasons of consistency and to enable the envisaged comparisons between approaches, we consecutively excluded respondents who had: a SLE point estimate lower than the current age (*n* = 3); a SLE lower than 60 or higher than 100 (because we did not have SSPs for those ages) (*n* = 37); provided the same answers to all five SSP questions (*n* = 25), including 19 respondents reporting a 50 % chance to all five target ages; an increasing SSP for higher ages (*n* = 24); or a distribution of SSP answers that did not intersect 50 % within the 60–100 years age range (*n* = 52). Finally, 737 respondents (60.3 %) remained for further analyses. Compared to the initial sample of 1223, this led to slightly more centred distributions for age and education and an underrepresentation of men. The sample characteristics are shown in Table 1.

**Table 1:** Sample characteristics (*n* = 737)

Variable	Category	Value
Age [Mean (SD)]		41.3 (11.3)
Age groups (%)	20–35 years	31.8
	36–59 years	68.2
Male (%)		47.6
Marital status (%)	Living alone/divorced	32.2
	Married/living together	67.8
Educational level (%)	Low	24.6
	Middle	44.9
	High	30.5
Income (%)	Low	28.1
	Middle	50.5
	High	21.4
(Self-) employed (%)		61.9
Having a severe disorder (currently/ever) (%)		26.5
Having a chronic disease (%)		35.8
Smoking (%)	Never	58.9
	Yes, occasionally	10.3
	Yes, daily	30.8
Kin’s age of death (%)	<75	21.0
	75 to 85	54.4
	≥85	24.6

Respondents were categorized into two age groups for further analyses because inspection of descriptive statistics of SSPs in different age groups showed a clear difference in SSPs between respondents aged below and above 35 years

Education: ‘Low’ = primary or secondary education; ‘Middle’ = upper secondary education or post-secondary non-tertiary education; ‘High’ = bachelor, master, doctoral or equivalent

Income (net household monthly income): ‘Low’ < €1500; ‘Middle’ = €1500–2999; ‘High’ = >€3000

**Fig. 1:**
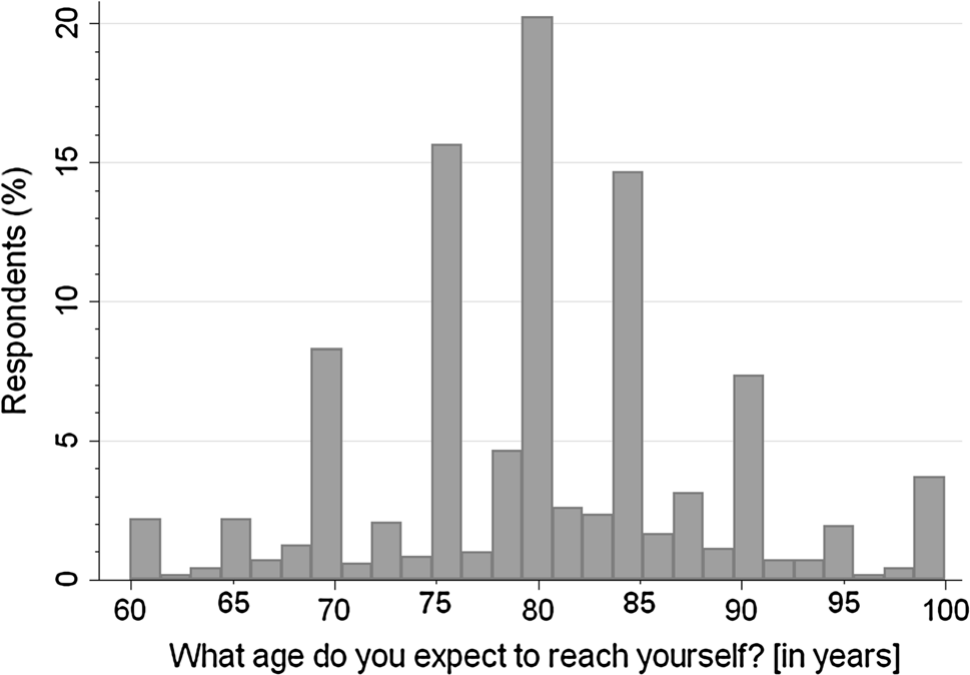
Distribution of the SLE point estimate (*n* = 737)

Using point estimates, mean SLE was 80.2 years (SD = 8.3). Figure 1 shows the frequency distribution of SLE point estimates. In line with earlier studies, approximately 40 % of answers were rounded to tens, and 70 % to fives. Peaks were observed at ages 75, 80 and 85.

**Fig. 2:**
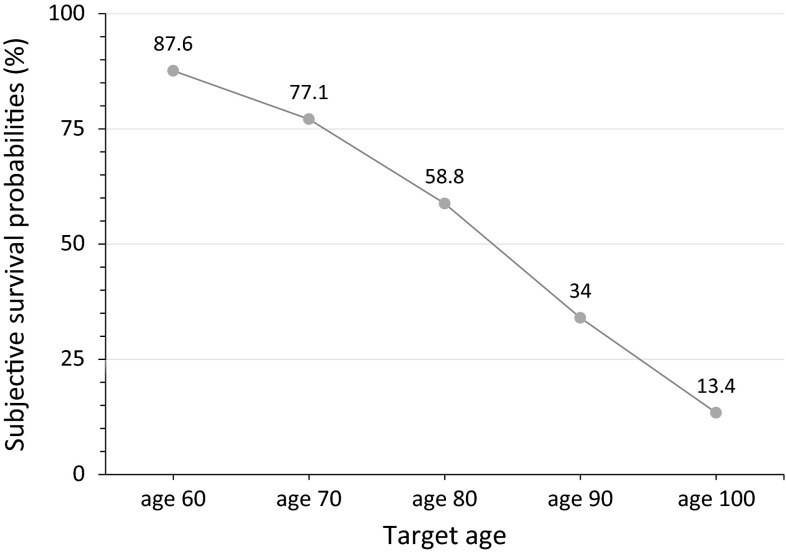
Subjective survival probabilities at target ages (*n* = 737)

Mean SSP declined from 87.6 % (SD = 13.6) at target age 60 to 13.4 % (SD = 15.5) at target age 100 (see Fig. 2). More than 75 % of responses to the five probability questions were multiples of ten, while almost 95 % were multiples of five. A “50 %” answer was most often observed for the SSP questions at target ages 80 and 90 (around 18 % of responses for both ages).

The mean point estimate obtained from the SSPs was 83.6 (SD = 9.3), which is on average 3.4 years (SD = 8.7) higher than the SLE point estimate. The SLE point estimate and the point estimate obtained from SSPs were correlated (*r* = 0.52, *p* < 0.001).

**Fig. 3:**
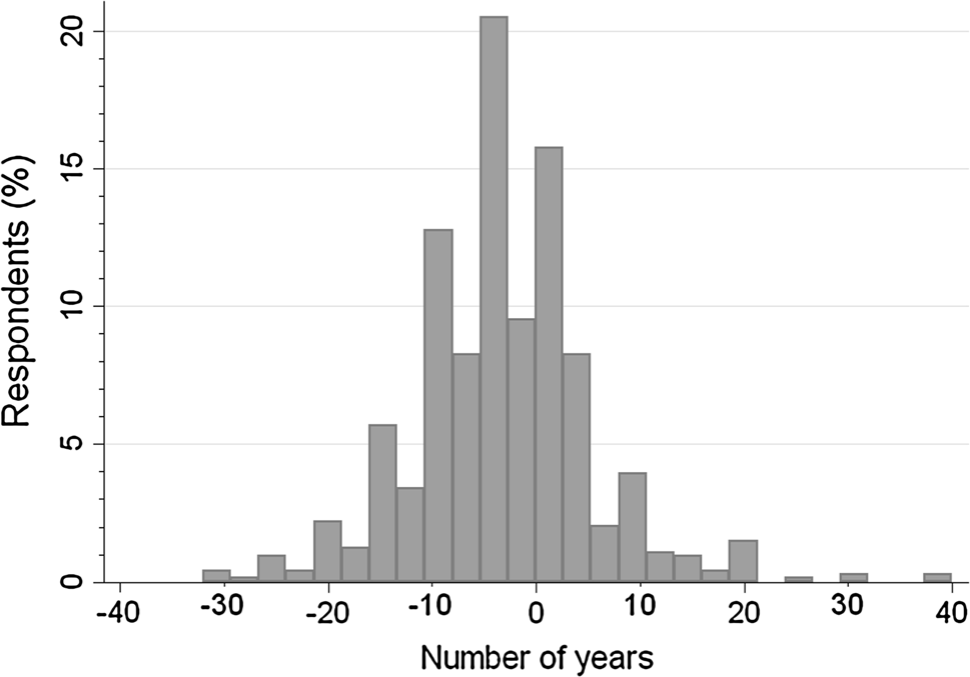
Distribution of differences between SLE and SSP point estimate (*n* = 737)

Individual differences between SLE and SSP point estimates ranged from −32 to +40 (Fig. 3), and the distribution showed a slight positive skew. Finally, the certainty score for the SLE point estimate derived from SSPs was 58.8 %.

### Variables associated with SLE and SSP

Table 2 shows the results of OLS regression models investigating variables associated with SLE and SSP point estimates, the difference between the two estimates, and the uncertainty surrounding the SLE point estimate.

**Table 2:** OLS regression analysis

Variables	SLE point estimate	SSP point estimate	Difference between SLE and SSP point estimates	Certainty score for SLE point estimate from SSPs
	(model 1)	(model 2)	(model 3)	(model 4)
Male	−0.54	0.25	−0.80	1.95
	(0.587)	(0.673)	(0.663)	(1.372)
Age group >35 years	−0.73	0.60	−1.33*	3.97***
	(0.659)	(0.743)	(0.747)	(1.492)
Low education	−0.42	−0.45	0.03	2.55
	(0.741)	(0.896)	(0.896)	(1.820)
High education	−0.42	−1.24*	0.83	−2.59*
	(0.647)	(0.741)	(0.686)	(1.531)
Low income	−0.32	−0.78	0.45	−0.40
	(0.683)	(0.781)	(0.785)	(1.628)
High income	0.75	−1.30	2.06***	−3.55**
	(0.689)	(0.841)	(0.779)	(1.664)
Kin’s age of death low	−4.64***	−4.79***	0.15	1.73
	(0.729)	(0.909)	(0.811)	(1.703)
Kin’s age of death high	4.18***	3.43***	0.76	−0.32
	(0.715)	(0.765)	(0.805)	(1.703)
Chronic disease	−1.88***	−1.05	−0.83	1.80
	(0.694)	(0.785)	(0.802)	(1.661)
Severe disorder	−1.53*	−1.63*	0.09	−0.56
	(0.794)	(0.845)	(0.883)	(1.844)
Smoking	−1.88***	−1.44**	−0.44	−0.16
	(0.628)	(0.717)	(0.700)	(1.431)
Constant	82.74***	85.49***	−2.75***	55.44***
	(0.753)	(0.904)	(0.913)	(1.762)
Observations	737	737	737	737
*R* ^2^	0.19	0.11	0.03	0.04
Adj. *R* ^2^	0.17	0.10	0.01	0.03

Robust standard errors in parentheses

*** *p* < 0.01, ** *p* < 0.05, * *p* < 0.10

The regression models for SLE (model 1) and SSP (model 2) showed similar outcomes. We found statistically significant associations with expected signs for severe disorder, smoking and age of death of next of kin. Having a chronic disease was only significant in the SLE model, (high) education only in the SSP model. Overall, the SLE model performed slightly better in terms of adjusted *R*-squared.

The difference between the SLE and SSP point estimates was associated with age and income (see model 3). The SSP point estimate was closer to the SLE point estimate for younger respondents and those with higher incomes.

The fourth model showed that the certainty score for the SLE point estimate was closer to the 50 % mark for respondents with higher education, higher income, and younger respondents.

## Discussion

In this short note, we presented estimations of subjective life expectancy based on two elicitation techniques, using a representative sample of the Dutch population aged 18–65 years in terms of age, gender and education level. On average, respondents were more optimistic (about 3.5 years) about their longevity when expressed in survival probabilities, using the 50 % chance point to calculate a SSP point estimate. Despite this difference, variables associated with SLE and SSP point estimates were very similar and their coefficient signs were plausible. Gender, age and socioeconomic variables like education and income were not strongly associated with the SLE and SSP point estimates. We found that age turned insignificant after introducing health indicators in the SLE model (results not shown here), which is not uncommon [7]. SLE and SSP point estimates were more similar for younger respondents and respondents with a higher socioeconomic status. This may reflect a higher capability of handling probability scores.

Some limitations of this study and the methods used are noted before highlighting the implications of our findings. First, this study was web-based and performed in one single country. This limits its generalizability. Second, excluding respondents with inconsistent answers from further analyses may have induced a selection bias in our results. Excluded respondents more often had a lower income and were male.

Nonetheless, we emphasise some important findings. First, inconsistencies in survival probabilities across target ages (i.e. same answers to all five SSP questions, increasing SSP for higher ages) were quite common (*n* = 49). Inconsistencies in SLE estimates (i.e. lower SLE than their current age) were less common (*n* = 3). Obviously, besides the difficulty of SSP estimates, this may reflect the fact that respondents answered five SSP questions but only one SLE question, providing greater opportunity for inconsistencies.

Second, rounding and focal point answers were common, as observed before [11, 12]. One in five respondents reported a SLE point estimate of exactly 80 years, for instance. While this may reflect a genuine expectation, it may also emanate from uncertainty, imprecision, or a tendency to provide focal answers. SSP responses also showed clear rounding issues. Here, special attention is required for a “50 %” answer. Bruine de Bruin et al. [20] for example, suggested that such “50/50” answers may indicate high uncertainty (similar to “donʼt know”) rather than a genuine probabilistic belief. Respondents reporting a 50 % chance for all five target ages (*n* = 19) were excluded from the analyses in this paper. Therefore, we expect that the remaining 50 % answers are more likely to represent a genuine probabilistic belief than high uncertainty, and thus to contain valuable information. Nevertheless, given that the SSP point estimate was determined using the probability of 50 %, this deserves more attention in future studies. Adjusting for probability weighting [21] may also be important.

**Fig. 4:**
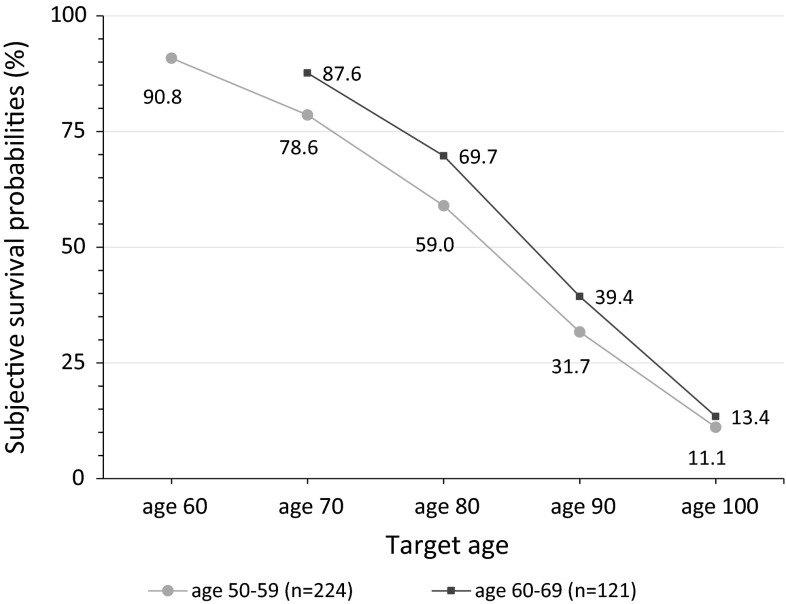
Comparison of SSPs between respondents aged 50–59 and 60–69

Third, sequential questioning may lead to anchoring [22]. Here, the probability of reaching the first target age given by respondents may have influenced probabilities at subsequent target ages. We tested this by comparing SSPs of respondents aged 50–59 (included in the current sample) with those of respondents aged 60–69 (excluded from the current sample). The younger group of respondents started with 60 as first target age, the older group with 70 as first target age. Interestingly, the answers of both groups resulted in very similar probability distribution curves, starting at almost the same probability, but the latter starting 10 years later (Fig. 4). While this may relate to a rational shift of expectations, it may also signal anchoring.

Our results relate well to existing literature. For instance, the explanatory variables significantly associated with subjective life expectancy were largely in line with those reported by Hamermesh [13]. Moreover, the difference found between the two methods (probability estimates being higher than point estimates) was in the same direction as reported by Hamermesh [13], albeit somewhat larger. This may relate to methodological differences between the studies (e.g., Hamermesh [13] used unrepresentative samples from the US, in which academic economists and male respondents were overrepresented, two instead of five target ages, and a different method of deriving subjective survival curves). Inconsistencies between these elicitation formats were also observed by Wu et al. [14].^6^

In conclusion, an increasing amount of research aims to understand (the formation of) subjective longevity expectations and their relation to health behaviours and outcomes. Different elicitation methods are used across studies. The results of the current study suggest that findings may not be directly comparable across studies, especially in certain subgroups of the population. Future work may compare both approaches in relation to objective survival expectations and predicting economic choice behaviour. More research on how to measure subjective expectations is therefore warranted.

## Footnotes

1 Verbal expectations questions (e.g. “How likely do you think it is that you will live up to 80 years old?”— very likely, fairly likely, not too likely, or not at all likely) are not within the scope of this short note.

2 First warm-up question: “Later on we will ask you what you think your chances are of reaching a certain age. Let us start with an example question about the weather. What are the chances that it will be a sunny day tomorrow? If you answer 90, this means that the chance that tomorrow will be a sunny day is 90 %. You can answer the following questions using a number between 0 and 100” (mean = 43.4; SD = 26.0; range 0–100). Second warm-up question: “Now an example about health. What are the chances that you will have a severe illness in the next 10 years?” (mean = 34.2; SD = 22.9; range 0–100).

3 If a respondent reported a probability of 50 % at one of the target ages, then that target age equalled the computed life expectancy based on SSPs (hereafter SSP point estimate). If a respondent answered 50 % at subsequent target ages, then the mean of those target ages was the SSP point estimate. If the probability of 50 % fell between the SSPs at two subsequent target ages, we employed linear interpolation to obtain the SSP point estimate.

4 If a respondent gave 90 years as point estimate of SLE, then the SSP the respondent gave for target age 90 (e.g. 70 %) was used as certainty score for the point estimate. If a respondent gave 85 years as point estimate for SLE, we employed linear interpolation of the SSPs for target ages 80 and 90 to obtain the certainty score for this point estimate.

5 A small group of respondents aged 18 and 19 years (*n* = 43) were excluded to form four equal age groups (20–29, 30–39 years, etc).

6 Note that the methodological approach applied by Wu et al. [14] differs from the current study as well. For example, Wu et al. [14] used a sample from a different country including respondents aged higher than in our study sample. Furthermore, they used a different approach to elicit SSPs. Respondents chose probabilities from a discrete list with ten categories representing a range of probabilities.
